# Sensitivity to Haptic Sound-Localization Cues at Different Body Locations

**DOI:** 10.3390/s21113770

**Published:** 2021-05-28

**Authors:** Mark D. Fletcher, Jana Zgheib, Samuel W. Perry

**Affiliations:** 1 Faculty of Engineering and Physical Sciences, Institute of Sound and Vibration Research, University of Southampton, University Road, Southampton SO17 1BJ, UK; 2 University of Southampton Auditory Implant Service, Faculty of Engineering and Physical Sciences, University of Southampton, University Road, Southampton SO17 1BJ, UK; JZ2G18@southamptonalumni.ac.uk

**Keywords:** cross-modal, cochlear implant, electro-haptic stimulation, haptic sound-localization, hearing aid, hearing impaired, neuroprosthetic, somatosensory, tactile, vibrotactile

## Abstract

Cochlear implants (CIs) recover hearing in severely to profoundly hearing-impaired people by electrically stimulating the cochlea. While they are extremely effective, spatial hearing is typically severely limited. Recent studies have shown that haptic stimulation can supplement the electrical CI signal (electro-haptic stimulation) and substantially improve sound localization. In haptic sound-localization studies, the signal is extracted from the audio received by behind-the-ear devices and delivered to each wrist. Localization is achieved using tactile intensity differences (TIDs) across the wrists, which match sound intensity differences across the ears (a key sound localization cue). The current study established sensitivity to across-limb TIDs at three candidate locations for a wearable haptic device, namely: the lower tricep and the palmar and dorsal wrist. At all locations, TID sensitivity was similar to the sensitivity to across-ear intensity differences for normal-hearing listeners. This suggests that greater haptic sound-localization accuracy than previously shown can be achieved. The dynamic range was also measured and far exceeded that available through electrical CI stimulation for all of the locations, suggesting that haptic stimulation could provide additional sound-intensity information. These results indicate that an effective haptic aid could be deployed for any of the candidate locations, and could offer a low-cost, non-invasive means of improving outcomes for hearing-impaired listeners.

## 1. Introduction

A cochlear implant (CI) is a neuroprosthesis that restores hearing by electrically stimulating the cochlea. While sound information is transmitted to the brain by thousands of hair cells in normal-hearing individuals, in CI users it is transmitted through a maximum of just 22 micro-electrodes. The amount of information that can be transferred is therefore severely limited. Despite this, CIs have been remarkably effective at restoring speech perception in quiet listening environments [1]. However, CI users often struggle to understand speech in noisy environments [2,3,4] and to locate sound sources [5,6]. Recent work has shown that CI listening can be enhanced using “electro-haptic stimulation” [3], whereby the electrical CI signal is augmented with haptic stimulation, which delivers sound information that the CI is unable to provide (reviewed in [7,8]). Using haptic stimulation on the wrists, substantial improvements to both speech-in-noise performance [3,4,9] and sound localization [6,10] have been shown. Haptic sound-localization accuracy was found to be better than for many hearing-aid users, suggesting that it could benefit a wide range of hearing-impaired listeners. Furthermore, the approach could be readily deployed using a compact, low-powered wearable device. As well as improving outcomes for CI and hearing-aid users, this device could aid the many millions of people worldwide who are unable to access hearing-assistive devices as a result of insufficient healthcare provision or prohibitive costs [7,11,12].

In haptic sound-localization studies, the audio signal received by behind-the-ear devices is converted to vibrotactile stimulation on the under (palmar) side of each wrist [6,10]. This allows the spatial-hearing cues used by the auditory system to be exploited. The dominant cues for sound localization are intensity and time differences across the ears, which are delivered as intensity and time differences across the wrists. It has recently been shown that across-limb sensitivity to intensity differences, but not time differences, is extremely high at the palmar wrist [13]. This strongly suggests that haptic sound-localization is achieved using tactile intensity differences (TIDs). This high sensitivity to across-wrist TIDs was also shown to be present across a large range of stimulus intensities, and to be similar for amplitude-modulated stimuli (like those typically used for electro-haptic stimulation [3,4,6,9,10]) and unmodulated stimuli. Importantly, it was also shown that TID sensitivity at the wrist does not decline with age (up to at least 60 years). These previous studies have all delivered haptic stimulation at the palmer wrist, but none have established whether this is the most suitable site for delivering haptic stimulation in the real world.

**Figure 1:**
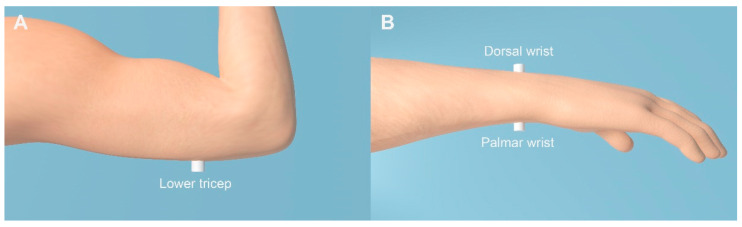
Illustration of the tactile stimulation probe placements that were used in the current study: lower tricep (panel (**A**)) and dorsal and palmar wrist (panel (**B**)).

The first aim of the current study was to compare sensitivity to across-limb TIDs for three candidate body locations at which a haptic device could be worn (see Figure 1). One potential issue with the palmar wrist location used in previous studies is that users often lean the underside of their wrists on surfaces (such as a table when typing at a computer). This could substantially alter the coupling of the haptic motor with the skin. An increased pressing force is known to improve tactile discrimination thresholds [14]. Uneven pressure across the wrists might therefore alter the perceived relative intensity across the wrists and distort haptic sound-localization cues. Another potential issue is that a number of devices deliver sound frequency information through differences in the location of stimulation on the skin, including at different points around the wrist [7,8,15,16,17]. If sensitivity to across-wrist TIDs changes with position around the wrists, then this might lead to different haptic sound-localization accuracy for sounds with different frequency content. Modern haptic drivers are able to calibrate motors based on the pressing force applied to them. However, although methods for continuously adapting to the amount of pressure applied are being developed, these techniques are not currently implemented on commercially available haptic drivers [18]. In addition to changing the pressing force, the coupling of the haptic motor with a surface could cause it to become substantially more audible, the pressing of the motor into the skin could cause discomfort, and an extruding object on the underside of the wrist could interfere with grasping actions. To address these issues, we compared across-wrist TID sensitivity on the palmar wrist to an alternative site on the back of the wrists (dorsal).

The wrist is commonly selected as a practical body location for haptic devices because wrist-worn devices allow for an adequate design space (in terms of size and weight), do not typically impede everyday tasks, and are easy to self-fit [7,19]. One downside of this is that the user’s wrist may already be engaged by another device (such as a fitness or health tracker or a smartwatch), leaving limited scope for an additional wrist-worn haptic aid for hearing to be fitted. It might therefore be desirable to integrate this approach into existing wrist-worn devices. Another potential issue is the discreetness of wrist-worn devices, which could become a particular concern if wrist-worn haptic aids to hearing were larger than other devices or were distinctive enough to become recognizable as aids for hearing. Finally, for haptic sound-localization devices, across-wrist TIDs might be distorted by wrist movements in everyday life (particularly crossing of the wrists), such as when walking, gesticulating, or operating machinery. To overcome these issues, we also tested TID sensitivity across the lower triceps, which allow for easy self-fitting, but are more discrete and less susceptible to distortion from arm movements.

For haptic sound-localization to be effective at enhancing performance in hearing impaired listeners, sensitivity to TIDs must be better than that available auditorily. For normal-hearing listeners, the smallest intensity difference between the ears that can be detected is around 1 dB [20]; for hearing aid users, this increases to ~2–3 dB [21]; and for CI users with implants in both ears, it increases further to ~4 dB [22]. However, it should be noted that variance between hearing-impairment listeners is high and that most CI users are implanted in only one ear, and so have little or no access to intensity differences between the ears [23]. Therefore, even if the across-limb TID discrimination was found to be 4 dB, this would still indicate that haptic stimulation could be effective at providing sound-localization cues to the majority of CI users.

The second aim of the current study was to establish the usable dynamic range available at each of the three candidate haptic device locations. Following previous work [13], the dynamic range is defined here as the difference between the lowest detectable signal and the most intense signal that can safely be delivered for 2 h. A large dynamic range is critical to represent the full range of intensity differences across the ears for all possible sound locations. The largest intensity differences across the ears can be greater than 20 dB [24], but the dynamic range for electrical stimulation by a CI is around just 15 dB [25,26]. In contrast, the dynamic range of haptic stimulation at the palmar wrist or the fingertip is around 60 dB [13,27]. Given the increased dynamic range available through haptic stimulation, a key role, in addition to enhancing spatial hearing for CI users, is likely to be the provision of additional sound-intensity information. It is therefore critical to establish the dynamic range available at each candidate stimulation site.

As with TIDs, changes in the pressing force during activities such as typing might change the perceived intensity and the dynamic range available, and this is likely to be a particular issue for devices stimulating the palmar wrist. Furthermore, for devices that provide frequency information through stimulation at different locations on the skin, changes in the pressing force might also lead to a distorted perception of the relative intensity of sound in different frequency bands. These issues are expected to be substantially reduced at the dorsal wrist and lower tricep.

Most previous work has focused on the sensitivity of the fingertip and few studies have compared tactile detection thresholds across different body sites. Across three studies, the thresholds were measured at the fingertip, the palm, and the palmar forearm. The highest sensitivity was found at the finger and the lowest at the forearm [28,29,30]. However, the hand and fingertip are not considered suitable sites for the current application, as they are frequently engaged in everyday activities. Another study found that the bicep was less sensitive than the hand, but that it was more sensitive than the abdomen, back, or shoulders [31], which have been used in some previous haptic devices [32]. In addition to the reduced sensitivity, devices fitted on the torso can also raise difficulties with self-fitting and can lead to undesirable feelings of restrictedness [7].

In this study, for all three locations tested, we found a large dynamic range and extremely high sensitivity to across-limb TIDs, which was similar to intensity difference sensitivity across the ears in normal-hearing listeners. This indicates that any of the candidate body locations studied could be used to dramatically improve sound localization for a wide range of hearing-impaired listeners. These findings could also have substantial implications for the design of other wearable haptic systems. Haptic devices are currently being developed for a variety research, medical, military, and entertainment applications, but currently do not take advantage of the high sensitivity to across-limb TIDs. For example, wrist-worn haptic devices have been developed for remote control of research laboratory equipment [33] and to aid needle steering in brachytherapy [34]. Wrist-worn haptic systems have also been developed to aid the remote control of robots that have been deployed in areas that are too small or dangerous for humans to access [35], and to enhance immersion and interactions in virtual or augmented reality [19]. There is a strong potential for these systems to exploit the tactile system’s large dynamic range and high sensitivity to across-limb TIDs to enhance spatial perception.

## 2. Materials and Methods

### 2.1. Partipants

Twelve participants (6 males and 6 females, aged between 22 and 33 years, with an average of 22.6 years of age) took part in this experiment. Participants were recruited from the staff and students of the University of Southampton, and each participant was paid an inconvenience allowance of £10 per hour. Written informed consent was obtained from all of the participants. Participants reported no touch perception issues and had vibrotactile detection thresholds within the normal range (see Procedure), indicating no dysfunction of their tactile system. The participant characteristics are shown in Table 1.

### 2.2. Stimuli

**Figure 2:**
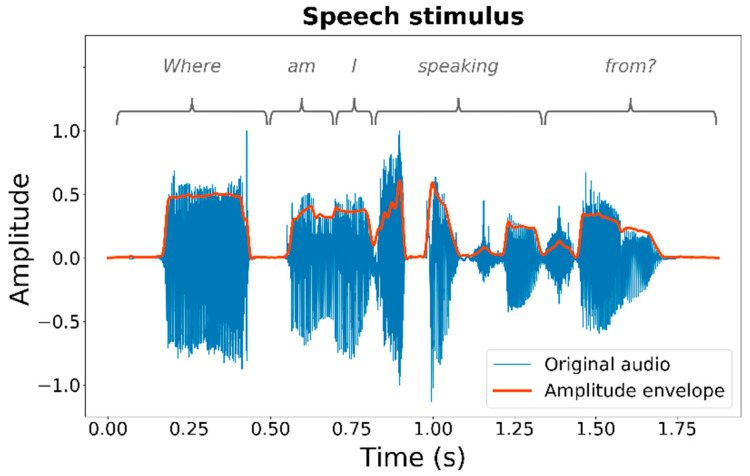
A speech sample and its amplitude envelope, which was used to modulate the amplitude of the tactile stimulus in the current study. The original audio waveform is shown in blue and the extracted amplitude-envelope is highlighted with a thick orange line. The spoken text is marked above the stimulus.

The tactile stimulus was a sinusoid with a frequency of 250 Hz, either with or without amplitude modulation applied. The modulated stimulus had its amplitude modulated by the amplitude envelope of a female speech sample (shown in Figure 2), which was used in previous studies [10,13]. The sample is available through the University of Southampton’s Research Data Management Repository at: https://doi.org/10.5258/SOTON/D1206 (last accessed on 27 May 2021). A 500th-order zero-phase FIR filter with a cut-off frequency of 50 Hz was applied to the absolute Hilbert transform of the speech sample to extract the amplitude envelope. The unmodulated stimulus had a duration of 600 ms (to ensure that maximal temporal summation had occurred [36]) and the modulated stimulus had a duration of 1877 ms (matching the duration of the speech sample). Stimuli were ramped on and off with 20-ms raised-sine and -cosine ramps.

The modulated stimulus was used in the TID discrimination threshold measurements. For these measurements, the stimulus intensity was nominally set to 20 dB above the individual’s modulated-stimulus detection threshold at the least sensitive wrist. The level of the stimulus was randomly roved (with a uniform distribution) around the nominal level by ±2 dB. This ensured that participants could not use single-wrist intensity cues to perform the task. All stimuli had total harmonic distortion of less than 0.1%. To mask any audio cues from the shakers, a white noise at a level of 65 dB SPL (A-weighted) was delivered to each ear.

### 2.3. Apparatus

During the screening phase, vibrotactile threshold measurements were made with a HVLab Vibrotactile Perception Meter (full details of the tactile vibrometer can be found at: [37]). A 6-mm contactor with a rigid surround and a constant upward force of 2N was used, following International Organization for Standardization specifications [38]. This system was calibrated using a B&K calibration exciter (Type 4294).

**Figure 3:**
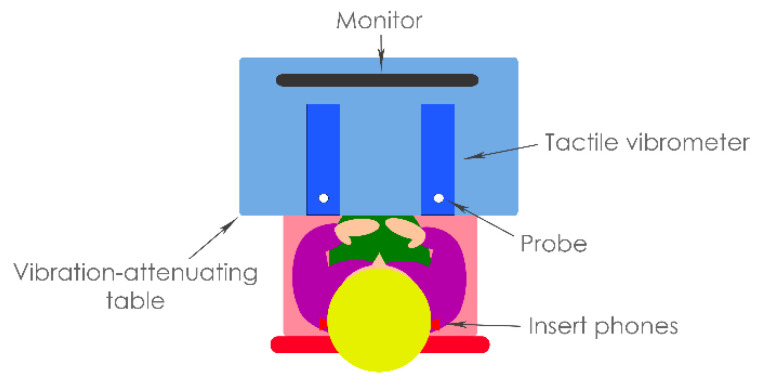
Schematic representation of the experimental set up for the testing phase, with the participant sitting in front of two tactile vibrometers and a computer monitor.

In the testing phase, detection threshold measurements were first conducted with stimuli controlled using a custom MATLAB script (version R2019a, The MathWorks Inc., Natick, MA, USA). The TID discrimination thresholds were then measured with stimuli controlled using a custom Max 8 patch (version 8.0.8, Cycling ‘74, Walnut, CA, USA). For all of the measurements in the testing phrase, an RME Babyface Pro soundcard (Haimhousen, Germany; sample rate of 48 kHz and bit depth of 24 bits) was used to play out haptic and audio signals. Haptic stimulation was delivered through two HVLab tactile vibrometers spaced shoulder-width apart (see Figure 3). These were mounted on vibration-attenuating foam in front of the participant. The vibrometers were adapted through the substitution of a standard 6-mm probe with a 10-mm circular probe. The probe size and shape were selected to match compact motors that are commonly used in haptic devices (e.g., [19]). The rigid surround was also removed to allow for an increased area of skin excitation, as would be typical of stimulation through a wearable haptic device. The vibration intensity was calibrated using the HVLab tactile vibrometer’s built-in accelerometers (Quartz Shear ICP, model number: 353B43), which were calibrated using a B&K Type 4294 calibration exciter.

Observation intervals were visually cued and feedback was given using a computer monitor (iiyama ProLite T2454MSC-B1AG 24-inch), placed 0.5 m in front of the participant. The masking noise was presented to each ear using ER-5 insert earphones (Etymotic, Chicago, IL, USA). Audio stimuli were calibrated using a B&K G4 sound level meter, with a B&K 4157 occluded ear coupler (Royston, Hertfordshire, UK). A B&K Type 4231 sound calibrator was used to calibrate the sound level meter.

### 2.4. Procedure

The study contained a screening and testing phase. The screening phase ensured that participants had normal touch perception. Participants were asked to complete a screening questionnaire to confirm that they (1) had not been exposed to hand or arm vibration in the previous 24 h, (2) had not been exposed to sustained periods of intense hand or arm vibration (e.g., working with heavy machinery) at any time, and (3) had no known conditions or injuries that might affect their touch perception (e.g., diabetes). Normal touch perception was also confirmed by measuring vibrotactile detection thresholds at the fingertip of the index finger on each hand, following International Organization for Standardization specifications [38]. To take part in the experiment, participant’s touch perception thresholds were required to be within the normal range (<0.4 ms^−2^ RMS at 31.5 Hz, and <0.7 ms^−2^ RMS at 125 Hz [38]). Finally, otoscopy was performed to ensure that there were no contraindications that would prevent safe earphone insertion. If participants passed all of the screening stages, they moved to the testing phase.

In the testing phase, vibrotactile detection and TID discrimination thresholds were measured at the center of the palmar and dorsal surface of the wrist and at the lower tricep (9 cm above the point of the elbow). The stimulation sites are illustrated in Figure 1. First, detection thresholds for both the modulated and unmodulated stimulus were measured separately for left and right limbs. Thresholds for the modulated stimulus were used to set the stimulus intensity in the TID discrimination threshold measurements and the thresholds for the unmodulated stimulus were used to estimate the dynamic range. A three-alternative forced-choice paradigm was used. Each trial comprised of three observation intervals, each separated by a gap of 200 ms. Only one interval (chosen at random with equal a priori probability) contained the signal and the task of each participant was to identify that interval. The timing of each interval was visually cued and, after each trial, feedback on whether the response was correct or incorrect was given. The stimulus intensity was varied using a two-down, one-up procedure. The stimulus intensity was initially set at 1.04 ms^−2^ RMS (frequency-weighting applied following British Standard 6842:1987 [39]). This was established in piloting to be an easily detected and comfortable intensity. For the first reversal of the adaptive track, the intensity was changed in 10 dB steps, for the second it was changed in 5 dB steps, and for the remaining six reversals it was changed in 2.5 dB steps. The threshold was estimated as the average of the final six reversals.

Following the detection threshold measurements, participants had a break of at least 15 min before beginning the TID discrimination threshold measurements. TID thresholds were measured for the modulated stimulus only. This stimulus mimicked the stimulus used in previous studies, which showed haptic enhancement of sound localization [6,10] and speech-in-noise performance [3,4,9] in CI users. Similar TID discrimination thresholds have previously been found for modulated and unmodulated stimuli [13]. For each participant and stimulation location, the stimulus intensity was set to 20 dB above the participant’s detection threshold for the modulated stimulus on the least sensitive limb. A two-alternative forced-choice paradigm was used, with the stimuli presented in two observation intervals for each trial. The first interval contained a reference stimulus, with no TID applied. The second interval contained the target stimulus, with a higher intensity on the left or right limb (with equal a priori probability). The participant was instructed to indicate to the experimenter whether the target stimulus was to the left or the right of the reference stimulus. As in the detection threshold measurements, intervals were visually cued and feedback was provided. The thresholds were measured using a three-down, one-up adaptive tracking procedure. The starting TID was 10 dB. TIDs were adapted by 2.5 dB for the first two reversals, by 1 dB for the third reversal, and by 0.25 dB for the remaining six reversals that made up a threshold track. A constant power panning law was used to calculate the intensity difference between the wrists. The gain applied to each side was determined using the following equation:

(1) $$g_{L}=\cos\left(\arctan\left(10^{\left(x/20\right)}\right)\right),$$

(2) $$g_{R}=\sin\left(\arctan\left(10^{\left(x/20\right)}\right)\right),$$

where x is the TID value in dB at the current point in the adaptive track, and the left and right shaker gains (in linear units) are $g_{L}\;$ and $g_{R}$, respectively. A positive value for x will lead to a higher intensity on the right, and a negative value will lead to a higher intensity on the left.

The threshold was defined as the average of the last six reversals. Three threshold tracks were run for each stimulation location and these were averaged to make the final threshold estimate.

Each experimental session lasted around 2 h. The order in which the stimulation locations (palmar wrist, dorsal wrist, and lower tricep) were measured was fully counterbalanced across participants. The experimental protocol was approved by the University of Southampton Faculty of Engineering and Physical Sciences Ethics Committee (ERGO ID: 47769). All of the research was performed in accordance with the relevant guidelines and regulations.

## 3. Results

**Figure 4:**
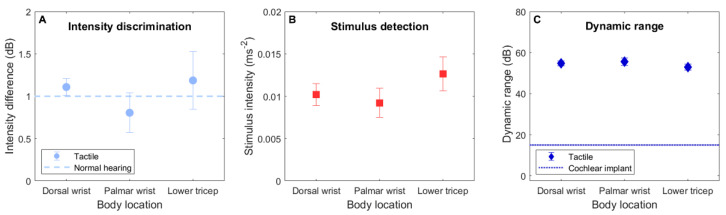
Panel (**A**): across-wrist tactile intensity difference thresholds for each body location. For reference, the dashed light blue line shows across-ear intensity difference discrimination thresholds in young normal-hearing adults (based on data from [20]). Panel (**B**): detection thresholds for the unmodulated stimuli at each location (averaged across left and right limbs). Panel (**C**): the usable dynamic range at each location (averaged across left and right limbs). The dotted dark blue line shows the dynamic range available through electrical CI stimulation (based on data from [25,26]). In all of the panels, the error bars show the standard error of the mean.

Figure 4A shows the TID discrimination thresholds for each body location. The TID discrimination data were analyzed using a repeated-measures analysis of variance (RM-ANOVA), with the factor “body location” (dorsal wrist, palmar wrist, and lower tricep). A significant effect of body location was found (*F*(2,22) = 6.16, *p* = 0.008). The average TID thresholds were 1.11 dB (ranging from 0.54 to 1.75 dB; SD = 0.34 dB) for the dorsal wrist, 0.81 dB (ranging from 0.58 to 1.08 dB; SD = 0.17 dB) for the palmar wrist, and 1.19 dB (ranging from 0.65 to 1.75 dB; SD = 0.37 dB) for the lower tricep. Planned post-hoc two-tailed paired-samples *t*-tests (with *p* values corrected for multiple comparisons using the Bonferroni–Holm method [40]) revealed that the TID discrimination thresholds for the palmar wrist were significantly lower than either for the dorsal wrist (*t*(12) = 3.41, *p* = 0.012) or the lower tricep (*t*(12) = 3.80, *p* = 0.009). No difference in the thresholds for the dorsal wrist and the lower tricep were found (*t*(12) = 0.53, *p* = 0.606).

A second RM-ANOVA was conducted to assess the detection threshold measurements (for the unmodulated stimulus), with the factors “side” (left or right) and body location. Figure 4B shows detection thresholds for each body location. No significant effect of side (*F*(1,11) = 0.40, *p* = 0.540) or body location (*F*(2,22) = 1.60, *p* = 0.225), or interaction between side and body location (*F*(2,22) = 0.82, *p* = 0.453) was found. For the dorsal wrist, the average detection thresholds for the left and right wrists were 0.0114 ms^−2^ (with frequency-weighting applied following BS-6842:1987 [39]; ranging from 0.0029 to 0.0203 ms^−2^; SD = 0.0057 ms^−2^) and 0.0091 ms^−2^ (ranging from 0.0029 to 0.0194 ms^−2^; SD = 0.0055 ms^−2^), respectively. For the palmar wrist, the average detection thresholds for the left and right wrists were 0.0104 ms^−2^ (ranging from 0.0018 to 0.0330 ms^−2^; SD = 0.0092 ms^−2^) and 0.0080 ms^−2^ (ranging from 0.0018 to 0.0203 ms^−2^; SD = 0.0060 ms^−2^), respectively. For the lower tricep, the average detection thresholds for the left and right wrists were 0.0116 ms^−2^ (ranging from 0.0024 to 0.0330 ms^−2^; SD = 0.0086 ms^−2^) and 0.0137 ms^−2^ (ranging from 0.0054 to 0.0330 ms^−2^; SD = 0.0091 ms^−2^), respectively.

Using the detection thresholds, the usable dynamic range was estimated for each body location. Following previous work [13], the dynamic range was defined as the difference (in dB) between the detection threshold and the maximum safe exposure (assuming no more than 2 h at the maximum exposure level per day [41]). Figure 4C shows the dynamic range calculated for each body location (averaged across left and right limbs). The average dynamic range for the dorsal wrist was 55.6 dB (SD = 4.3 dB), with the largest dynamic range for either limb being 65.7 dB and the smallest being 48.8 dB. The average dynamic range for the palmar wrist was 57.6 dB (SD = 6.5 dB), with the largest dynamic range being 69.9 dB and the smallest being 44.6 dB. The average dynamic range for the lower tricep was 54.3 dB (SD = 5.2 dB), with the largest dynamic range being 67.4 dB and the smallest being 44.6 dB.

Planned post-hoc two-tailed Pearson’s correlation analyses (with Bonferroni–Holm correction applied) revealed no correlation between the detection thresholds and TID discrimination thresholds (dorsal wrist: *r*(12) = −0.166, *p* ≥ 0.999; palmar wrist: *r*(12) = −0.289, *p* ≥ 0.999; lower tricep: *r*(12) = 0.0.27, *p* ≥ 0.999). Additional unplanned post-hoc paired-sample t-tests (with Bonferroni–Holm correction) were conducted to investigate whether there was an effect of sex on the detection thresholds and TID discrimination thresholds at each body location. No effect was found for the detection thresholds (dorsal wrist: *t*(5) = −2.043, *p* = 0.485; palmar wrist: *t*(5) = −0.966, *p* ≥ 0.999; lower tricep: *t*(5) = −0.284, *p* ≥ 0.999) or TID discrimination thresholds (dorsal wrist: *t*(5) = 0.461, *p* ≥ 0.999; palmar wrist: *t*(5) = 1.217, *p* ≥ 0.999; lower tricep: *t*(5) = 3.470, *p* = 0.108).

## 4. Discussion

The across-limb TID discrimination threshold was found to be around 1 dB for each of the body locations tested. Encouragingly, these thresholds were highly similar to those for the across-ear intensity difference discrimination in normal-hearing listeners [20]. This suggests that an even greater haptic sound-localization accuracy than has previous been shown [6,10] could be achieved. The palmar wrist, which has been used in previous studies of haptic sound-localization [6,10], was found to be more sensitive to TIDs than the dorsal wrist or lower tricep (which did not differ from each other). TID discrimination thresholds measured at the palmar wrist were in strong agreement with a previous study that assessed TID sensitivity at this body location [13]. While the higher sensitivity at the palmar wrist may indicate that it is the most suitable body location for delivering haptic sound-localization cues, it should be noted that the average difference in TID sensitivity between this and the other locations measured was relatively small (~0.3 dB). Furthermore, as discussed in the Introduction, the dorsal wrist or lower tricep may be preferred if issues such as contact with surfaces or crossing of the wrists are realized in practice.

To appreciate the sensitivity to across-limb TIDs demonstrated in this study, it is important to understand how the minimum detectable TID relates to the difference in sound location. However, this relationship is complex, as the TID extracted from across-ear intensity differences depends strongly on sound frequency, the starting location of the sound relative to the head, and the haptic signal-processing strategy used. Using the long-term average spectrum of male speech [42] and the haptic signal-processing approach from Fletcher and Zgheib [10], the minimum detectable TID across the palmar wrists corresponds to a change in stimulus location in the horizontal plane of ~ 3° for a starting location between ±15° (with 0° corresponding to directly in front of the listener); for a starting location between 15° and 30° to the side, it would correspond to a change of ~ 4.5°; and, for a starting location between 30° and 45° to the side, to a change of ~ 7°. This sound-localization accuracy is far higher than is typically achieved by hearing-impaired listeners [5,6].

At all candidate body locations, the average usable dynamic range was around 55 dB. Across participants, the smallest dynamic range for any of the body locations on the left or right side was 45 dB and the largest was 70 dB. Even the smallest dynamic range far exceeded that available through electrical CI stimulation [25,26], and also far exceeded the range required to represent the maximum intensity difference across the ears for any sound location [10,24]. These findings are in strong agreement with previous studies at the palmar wrist and fingertip [13,27]. Given the larger dynamic range available, as well as providing spatial hearing cues to hearing-impaired listeners, a haptic device could provide additional sound-intensity information (including absolute intensity) to CI users.

The current results showed considerable differences in detection threshold across stimulation sites, both within and across individuals. Because the frequency difference sensitivity of the tactile system is low [43], a number of haptic device prototypes have mapped sound-frequency information to haptic stimulation location along or around the wrist or forearm [7,15,16,17]. Individual correction for detection threshold differences (like that used in [10]) might therefore be desirable in haptic aids to hearing in order to avoid a distorted perception of frequency and sound location.

The secondary analysis showed no evidence of sex differences for the detection and TID-discrimination thresholds. Previous studies have shown evidence of sex differences in tactile detection thresholds at the wrist and in across-wrist TID discrimination [13]. There is also evidence that the tactile sensation magnitude grows slightly more quickly in women than in men [44], and that men are more sensitive to tactile time differences across the fingers than women [45]. However, further work with a large sample size is required for the presence or absence of sex differences to be securely established. An additional secondary analysis in the current study also revealed no evidence of a correlation between detection thresholds and TID discrimination thresholds, which is in line with previous findings [13].

The results of the current study strongly suggest that a haptic device deployed at any of the candidate body locations could effectively provide sound-localization information for a wide range of hearing-impaired listeners. A low-cost, non-invasive wearable haptic device could offer a highly attractive alternative to a second CI, which is expensive, limits access to future therapies and technologies, and risks vestibular dysfunction and loss of residual hearing. Such a haptic device could readily be developed using existing technologies [7].

In addition to aiding sound localization, there is evidence that the approach used in haptic sound-localization studies can substantially improve the segregation of spatially-separated sound sources [4]. If an inexpensive device that could aid sound localization and segregation were to be developed, then, in addition to helping many hearing-assistive device users, it could have a large impact for those unable to access hearing-assistive technology. This includes many millions of people in low- to middle- income countries [7,11,12], where unmanaged hearing loss often leads young children to have large deficits in educational attainment and in language and cognitive development [46,47,48,49,50]. Employment rates for hearing-impaired adults are also lower in these countries, and those who are employed tend to work in lower-grade occupations [50,51]. A new generation of haptic aids might reduce this burden by offering a low-cost and widely accessible means to improve access to the auditory world.

### 4.1. Limitations

In the current study, participants were not trained to discriminate across-limb TIDs. Across-ear intensity difference detection thresholds have been shown to improve with long-term training [52]. In this previous work, the majority of the improvement was observed after three 1 h training sessions, although improvement continued throughout the 9-h training regime. Substantial long- and short-term training effects have also been observed for haptic sound-localization [6,10] and for haptic enhancement of speech reception [3,9,53,54]. It therefore seems likely that an even greater sensitivity to across-limb TIDs than has been shown here can be achieved with training.

The current study used a stimulation frequency of 250 Hz and a circular contactor with a 10-mm diameter. These were selected to match the shape, size, and operating frequency of many widely available haptic actuators (e.g., [19]). A key reason that haptic actuators often deliver haptic stimulation at or close to 250 Hz is that tactile sensitivity is highest at this frequency [27]. For actuators that stimulate at 100 Hz (the lowest operating frequency typically found in compact actuators), detection thresholds are around 15 dB higher [27]. Contactor size is also known to impact detection thresholds, with thresholds increasing by around 3 dB with each halving in the contactor area [28,29]. Motors with different operating frequencies or smaller contactors would therefore be expected to have a reduced usable dynamic range. However, it should be noted that motors with a small contactor and low operating frequency would still typically have a usable dynamic range far larger than what is available through electrical CI stimulation.

### 4.2. Future Work

The main barrier to establishing the effectiveness of haptics for aiding spatial hearing in the real world is the absence of a suitable device. The technology required to build such a device is readily available, particularly after important recent advances in wireless communication, batteries, micro computation, 3D printing, haptic drivers, and haptic motors (see [7] for a review). Furthermore, many of the key technologies have already been combined in a compact form factor in the latest hearing aids and CIs, and some existing wearable haptic devices that could be adapted for this application are currently under development [7,16,17,19].

As well as developing the hardware for a haptic device, future work should explore whether more advanced haptic signal-processing strategies can be used to maximize benefits. Fletcher and Zgheib [10] recently proposed a haptic signal-processing strategy that included the use of dynamic-range compression. This compression was applied independently across frequency bands to reduce differences in sound energy across frequency. Across-ear intensity differences depend strongly on frequency [10,24] and, therefore, for a given sound location, stimuli with different distributions of energy across frequency can produce a different overall across-ear intensity difference. By reducing the difference in energy across frequency between sounds, this multi-band compression approach aims to make the correspondence between across-ear intensity difference and sound location more uniform between sound sources. In addition to exploiting techniques such as multi-band compression, the effectiveness of approaches that aim to improve sound localization by exaggerating across-ear intensity differences [55,56] should be tested for haptic sound-localization.

Techniques to improve sound segregation should also be explored. This includes sophisticated noise-reduction methods, such as those using neural networks [57,58], as well as simpler methods. For example, deploying a simple noise-reduction approach (using an expander) in a haptic signal-processing regime has been shown to be effective in allowing haptic stimulation to improve speech-in-noise performance in CI users [3,4,9]. Future work should explore whether this approach can also be effective for spatially-separated signal and noise sources.

In addition to allowing the transmission of spatial-hearing cues to aid listening, the high sensitivity to across-limb TIDs could be exploited in numerous other human–machine interfaces to deliver fine-grain orientation information. This could be beneficial in the applications already discussed, such as remote control of laboratory equipment [33], needle guidance [34], and remote human–robot control [35]. This high sensitivity might also be exploited for guidance in search and rescue missions where visibility is reduced [59], and to provide feedback on body orientation to those with balance disorders [60]. Future work should focus on establishing the effectiveness of using across-limb TIDs in other applications such as these.

## 5. Conclusions

The current study found a remarkably high sensitivity to across-limb TIDs for the palmar wrist, dorsal wrist, and lower tricep. Spatial hearing cues, presented through across-wrist TIDs, have been shown to substantially improve sound localization in CI users. The current findings suggest that an even greater sound localization accuracy than was previously measured could be achieved by haptic devices mounted at any of the body locations tested here. This approach could therefore be highly effective at enhancing spatial hearing for a wide range of hearing-impaired listeners. For each of the body locations tested, a large usable dynamic range was also found, which far exceeded that available through electrical CI stimulation. This large dynamic range will allow the tactile system to represent the full range of intensity differences between the ears that can occur at different sound source locations. It could also give valuable additional sound-intensity information to hearing-impaired listeners. A haptic device that improves spatial hearing in CI users could provide a low-cost, non-invasive alternative to the implantation of a second CI. In addition to aiding hearing-assistive device users, a low-cost haptic aid to hearing could be used to help the many millions of people worldwide who are currently unable to access hearing-assistive technology.

## Figures and Tables

**Table 1:** Participant characteristics. Participant age, sex (M = male, F = female), dominant hand (L = left, R = right), and vibration thresholds at the fingertip (measured during screening; see Procedure) are shown.

Part ID	Age (Years)	Sex	Dom Hand	Vib Thresh, 31.5 Hz, Right (ms^−2^ RMS)	Vib Thresh, 31.5 Hz, Left (ms^−2^ RMS)	Vib Thresh, 125 Hz, Right (ms^−2^ RMS)	Vib Thresh 125 Hz, Left (ms^−2^ RMS)
1	22	F	R	0.039	0.039	0.063	0.042
2	33	M	R	0.020	0.020	0.035	0.049
3	26	M	R	0.044	0.053	0.091	0.094
4	24	M	R	0.072	0.087	0.159	0.062
5	32	M	R	0.031	0.020	0.026	0.017
6	26	F	R	0.046	0.018	0.019	0.022
7	25	F	R	0.031	0.031	0.073	0.045
8	27	F	R	0.034	0.032	0.063	0.061
9	25	F	R	0.014	0.031	0.037	0.077
10	25	M	R	0.046	0.018	0.189	0.077
11	26	F	R	0.019	0.041	0.027	0.038
12	28	M	R	0.033	0.021	0.068	0.044
Mean	26.6	NA	NA	0.036	0.034	0.071	0.052

## Data Availability

All data supporting this study are openly available from the University of Southampton repository at: https://doi.org/10.5258/SOTON/D1838 (last accessed on 27 May 2021).
